# Supplementary material for: Caspase-resistant ROCK1 expression prolongs survival of Eµ-Myc B cell lymphoma mice
Source: Dis Model Mech. 2024 May 21;17(5):dmm050631. doi: 10.1242/dmm.050631 (PMC11139034; doi:10.1242/dmm.050631)
Supplement: Supplementary information [file dmm-17-050631-s1.pdf]

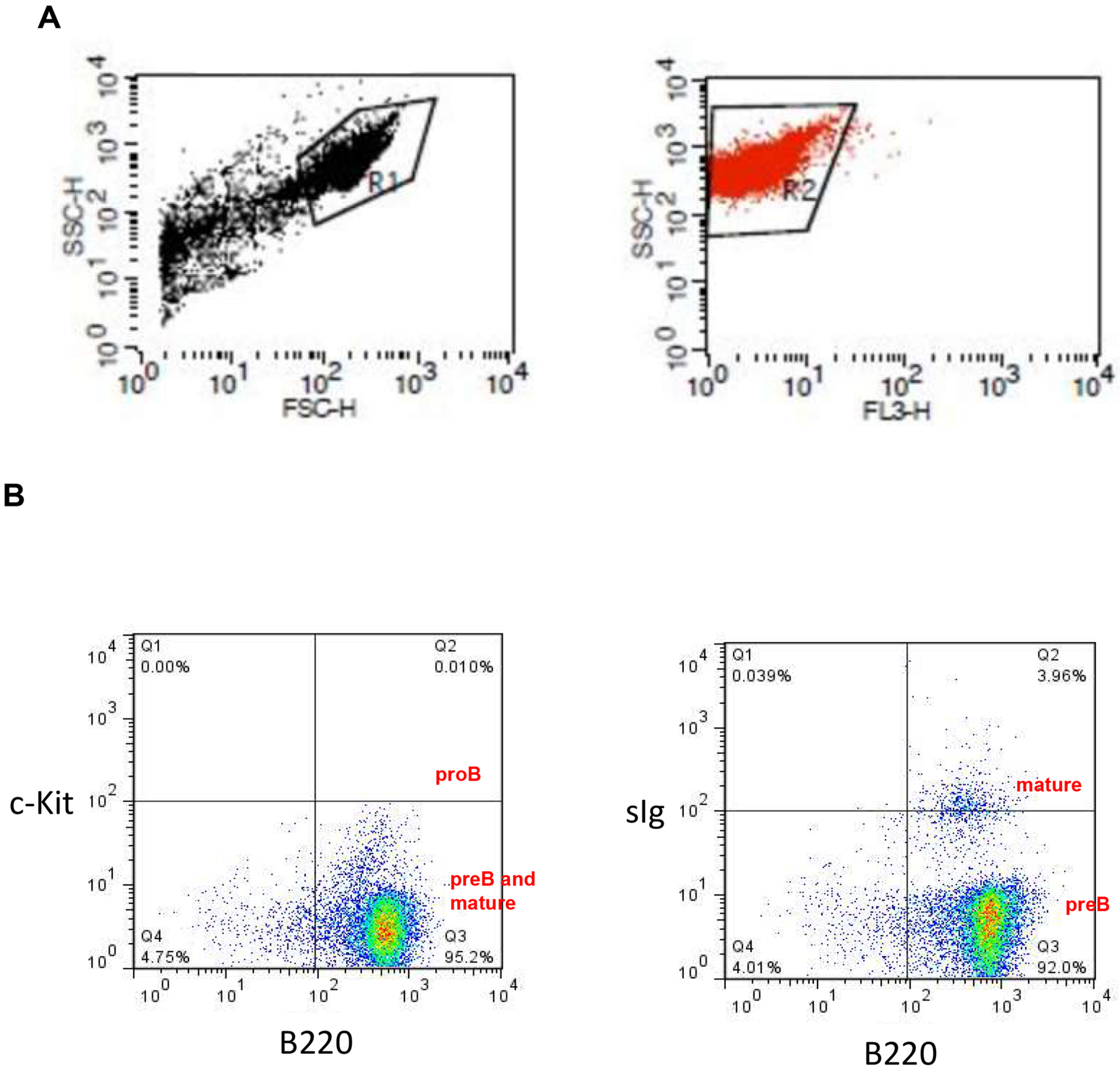

Fig. S1. Representative B cell immunophenotyping.

A. Initial gating schedule with exclusion of debris (left panel) and 7-AminoactinoMycin D (7AAD; FL3-H) incorporation by dead cells (right panel). **B.** Representative FACS plots to distinguish c-Kit^{+} B220 $^{+}$ pro-B cells from c-Kit^{-} B220^{+} pre-B and mature B cells (left panel), and B220^{+} cell surface IgG (sIg)^{low} pre-B cells from B220^{+} sIg^{high} mature B cells (right panel).

Disease Models & Mechanisms • Supplementary information

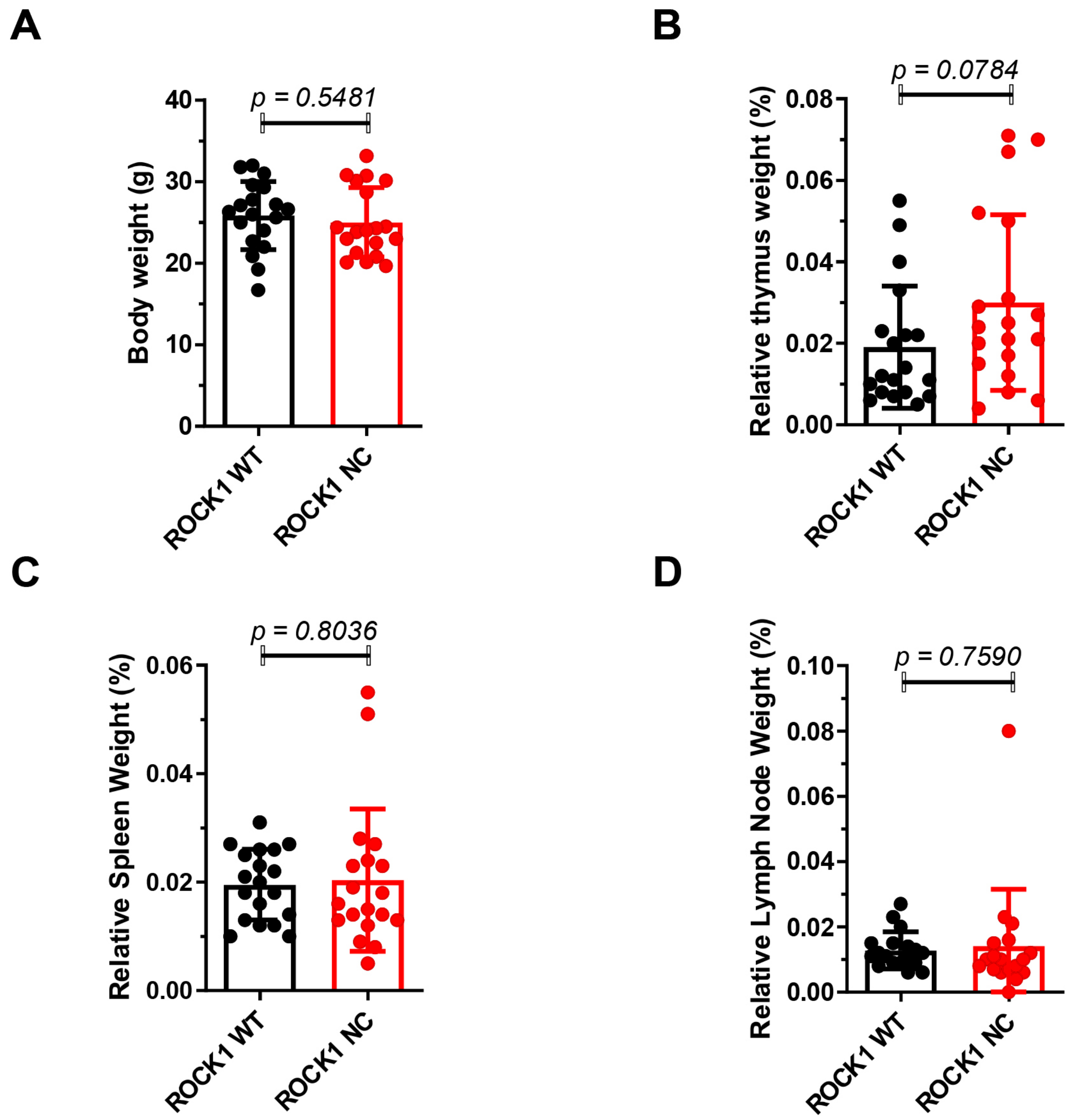

Fig. S2. Whole body, thymus, spleen and lymph node weights of *Eμ-Myc; Rock1 WT* and *Eμ-Myc; Rock1 NC* mice.

A. Weights of *Eμ-Myc; Rock1 WT* (n = 19) or *Eμ-Myc; Rock1 NC* (n = 19) mice at experimental endpoint. Relative weights compared to body weights of **B.** thymus, **C.** spleen and **D.** lymph node for each individual mouse. All *p* values were determined by unpaired Student's *t*-tests between indicated groups. All graphs show means ± standard deviation, with data points representing individual mice.

Disease Models & Mechanisms • Supplementary information

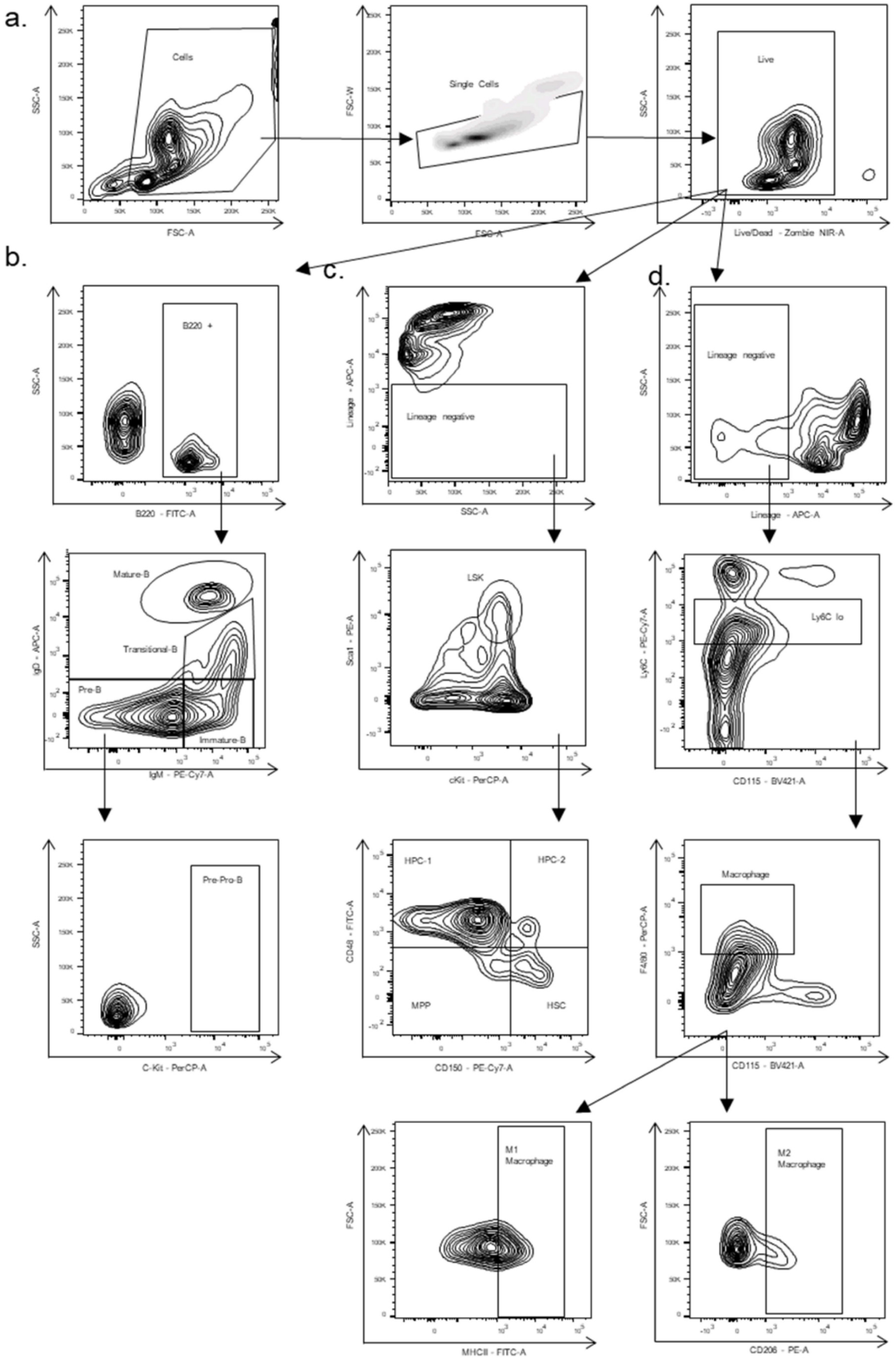

Disease Models & Mechanisms • Supplementary information

Fig. S3. Representative flow cytometry gating schedule for bone marrow cells.
A. Initial gating schedule with exclusion of debris (left), doublets (middle) and dead cells (right) Subsequent gating schedules shown (separate stain panel for each). **B**. B cell gating. **C.** Hematopoietic stem cell precursor gating. **D.** Macrophage gating.

Disease Models & Mechanisms • Supplementary information

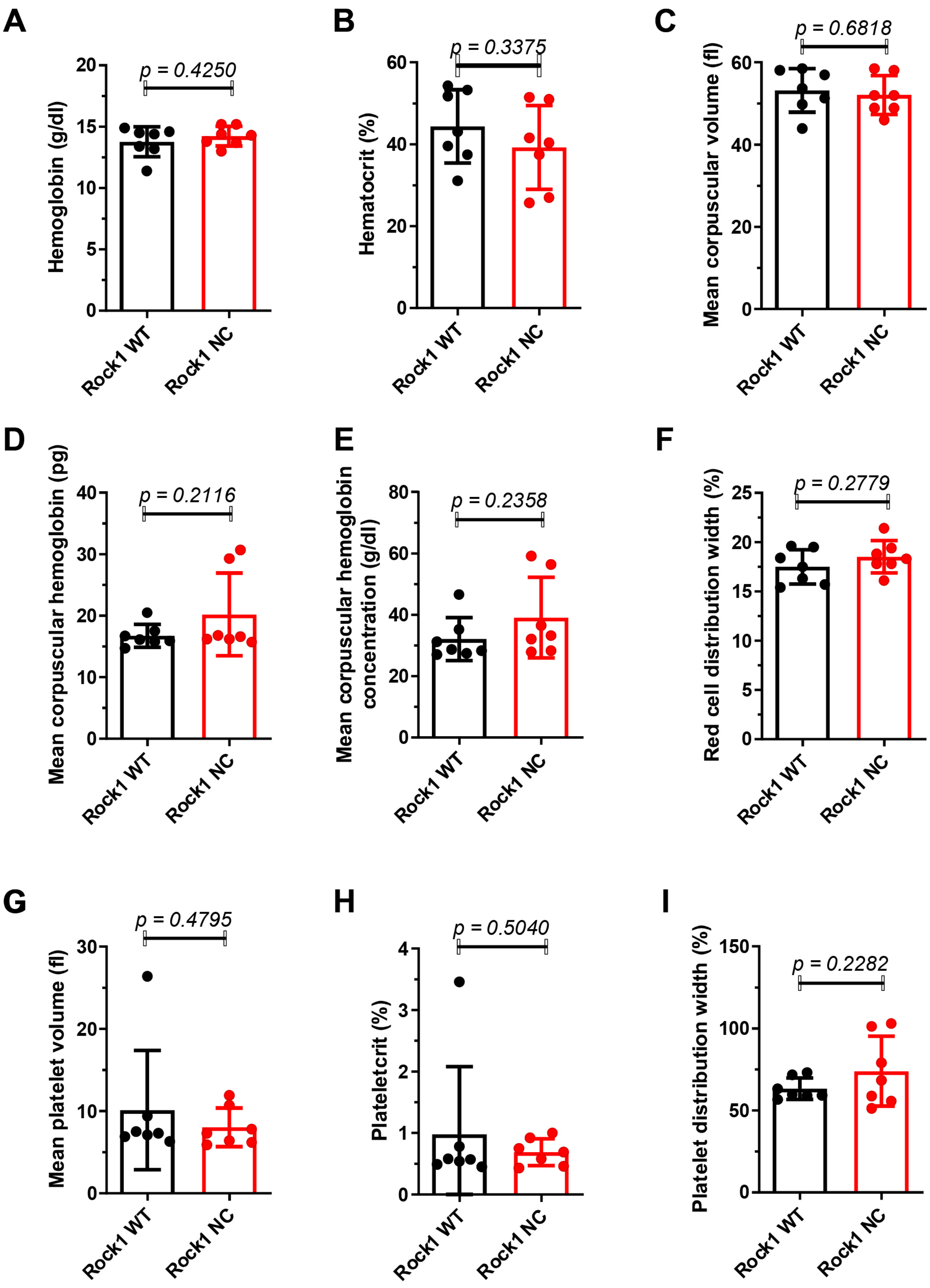
A
Hemoglobin (g/dl)
p = 0.4250
B
Hematocrit (%)
p = 0.3375
C
Mean corpuscular volume (fl)
p = 0.6818
D
Mean corpuscular hemoglobin (pg)
p = 0.2116
E
Mean corpuscular hemoglobin concentration (g/dl)
p = 0.2358
F
Red cell distribution width (%)
p = 0.2779
G
Mean platelet volume (fl)
p = 0.4795
H
Plateletcrit (%)
p = 0.5040
I
Platelet distribution width (%)
p = 0.2282
Rock1 WT
Rock1 NC

Fig. S4. Hematological parameters for pre-neoplastic Eμ-Myc; Rock1 NC and *Eμ-Myc; Rock1 NC* mice.

A. Hemoglobin, **B.** Hematocrit, **C.** Mean corpuscular volume, **D.** Mean corpuscular hemoglobin, **E.** Mean corpuscular hemoglobin concentration, **F.** Red cell distribution width, **G.** Mean platelet volume, **H.** Plateletcrit, and **I.** Platelet distribution width for *Eμ-Myc; Rock1 WT* (n = 7) or *Eμ-Myc; Rock1 NC* (n = 7) mice at experimental endpoint. All *p* values were determined by unpaired Student's *t*-tests between indicated groups. All graphs show means ± standard deviation, with data points representing individual mice ≤ 8 weeks of age.

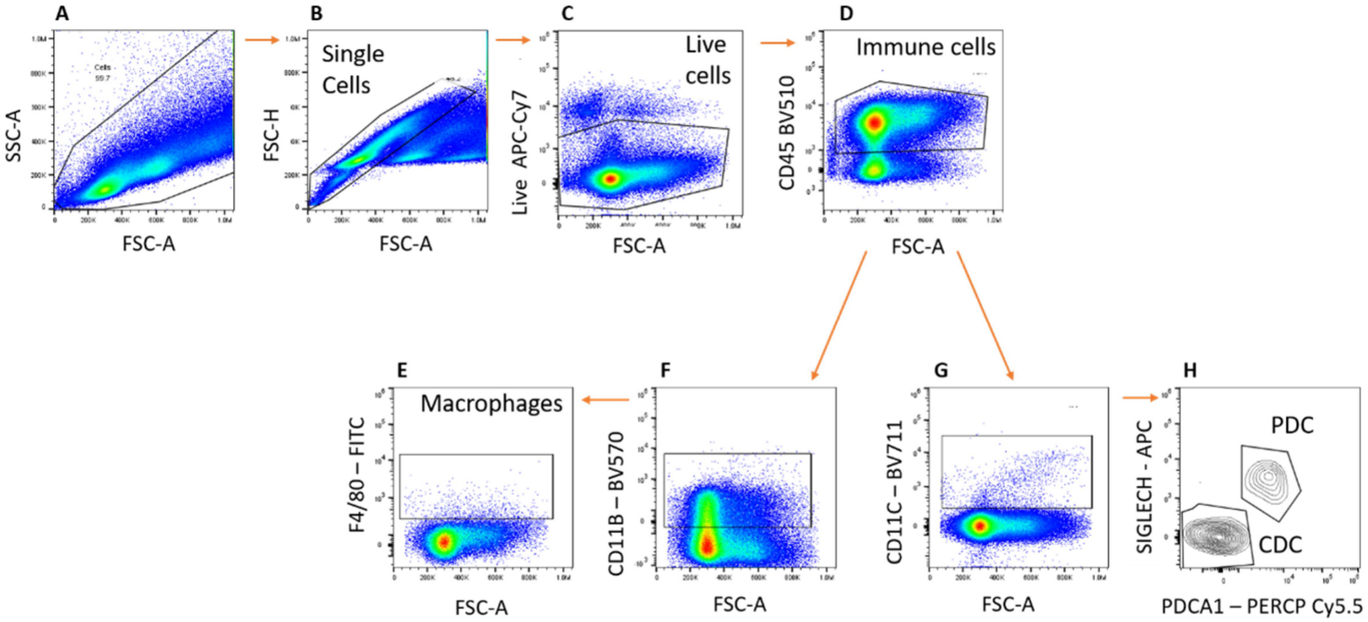

Fig. S5. Representative flow cytometry gating strategy for myeloid cell subtypes. All cells were gated in panel **A**, followed by a doublet exclusion in **B**. Gates were then applied on live cells in panel **C**. Cells were then selected for the CD45 immune cell marker in panel **D**. Cells were then divided based on their being CD11b positive (panel **F**) for macrophages or CD11c positive (panel **G**) for dendritic cells (DCs). CD11B$^+$ cells were then selected for F4/80 positivity (panel **E**). DCs were categorised into classical DCs (CDCs) or plasmacytoid DCs (PDCs) in panel **H**. PDCs are positive for both SIGLEC-H and PDCA-1. CDCs on the other hand are negative for both these markers. Cells were run through an Attune Flow Cytometer and analysed using FloJo.

Disease Models & Mechanisms • Supplementary information

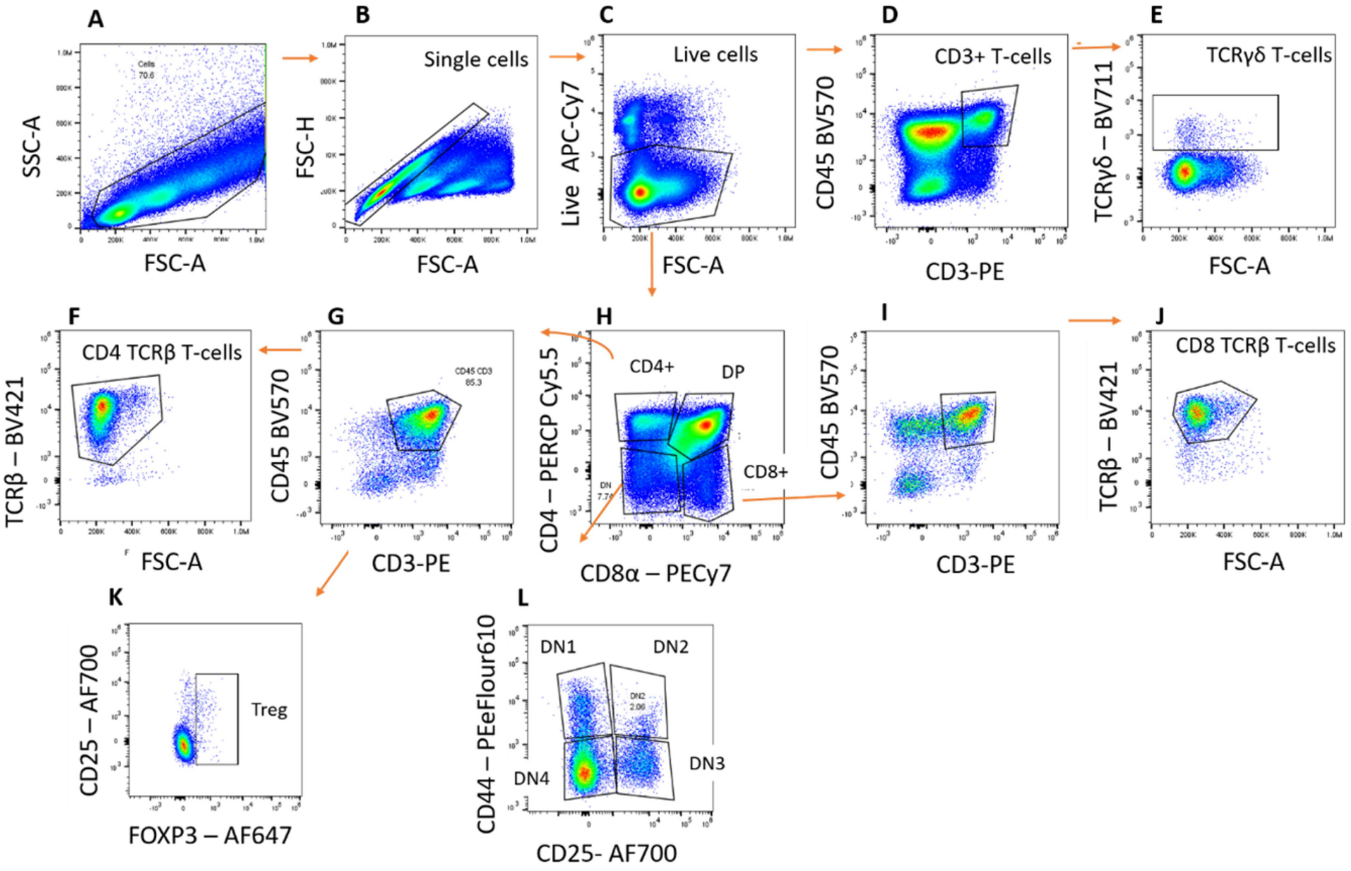

Fig. S6. Representative flow cytometry gating strategy for T-cell phenotyping.

All cells were gated in panel **A**, followed by a doublet exclusion in **B**. Gates were then applied on live cells in **C**. The γδ-T cells were then gated by selecting for cells that were double positive for CD45 and CD3 in panel **D**, followed by cells positive for the δγ T-cell receptor (TCR) in panel **E**. For the other cell types of interest, live cells were separated based on expression levels of CD4 and CD8 (**H**). Cells positive for both CD4 and CD8 are the double positive (DP) cells. Cells negative for CD4 and CD8 are the double negative (DN) cells. The double negatives (DN) were further subtyped based on CD25 and CD44 expression (**L**). These include the DN1 ($CD25^- CD44^+$), DN2 ($CD25^+ CD44^+$), DN3 ($CD25^+ CD44^-$) and DN4 ($CD25^- CD44^-$). The mature CD8 T-cells were gated based on CD8 positivity in panel **H**, followed by CD45 and CD3 positive in panel **I**, and finally by TCRβ positivity in panel **J**. Mature CD4 cells were gated on single positive CD4 cells in panel **H**, CD45 and CD3 positivity in panel **G**, and finally TCRβ positivity in panel **F**. Additionally, CD4 regulatory T cells (Tregs) could be identified by selecting for FOXP3 positivity in panel **K**, following selection in panel **G**. Cells were run through an Attune Flow Cytometer and analysed using FloJo.

Disease Models & Mechanisms • Supplementary information

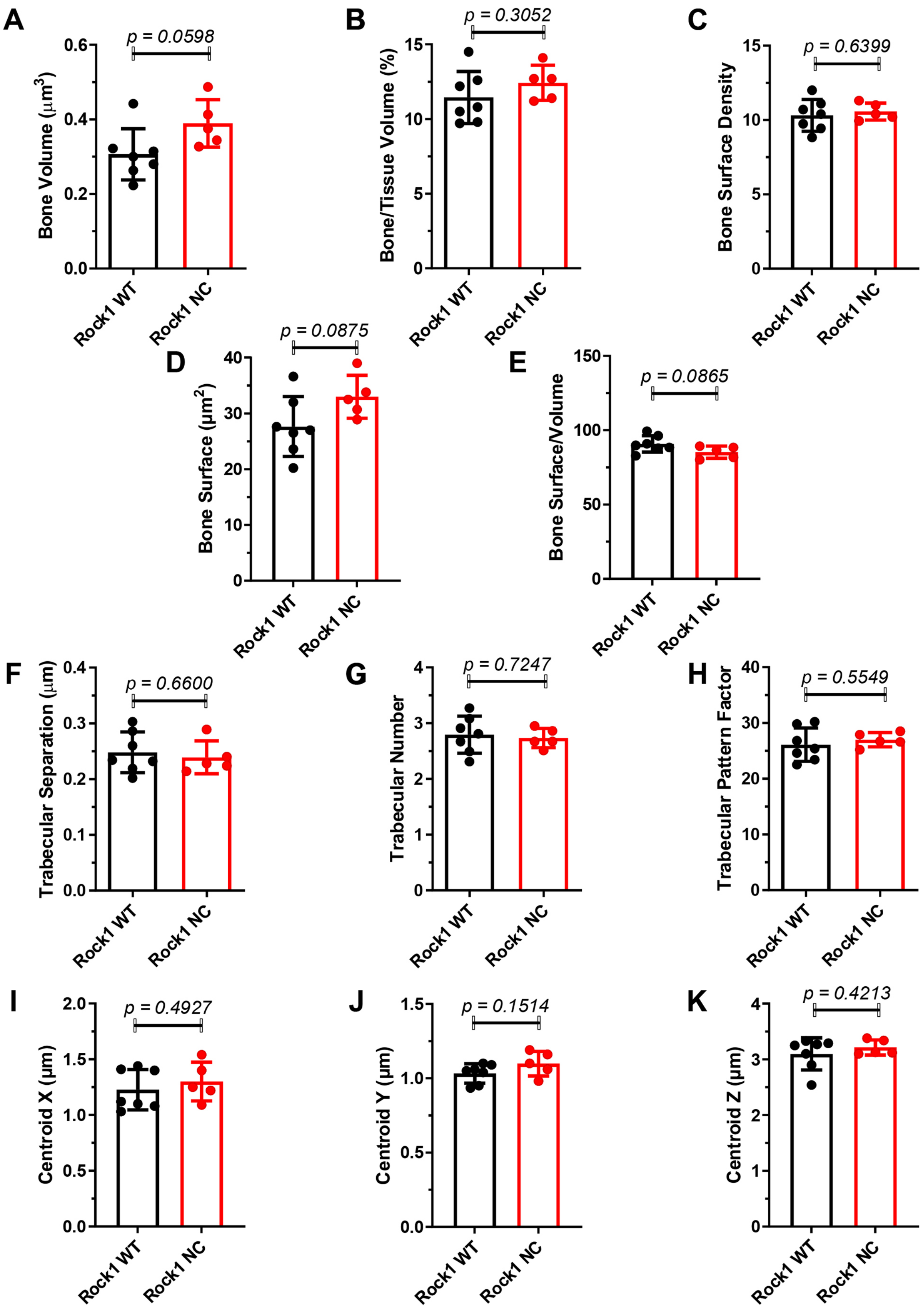
A
p = 0.0598
Bone Volume (μm3)
0.6
0.4
0.2
0.0
Rock1 WT
Rock1 NC
B
p = 0.3052
Bone/Tissue Volume (%)
15
10
5
0
C
p = 0.6399
Bone Surface Density
D
p = 0.0875
Bone Surface (μm2)
40
30
20
10
0
E
p = 0.0865
Bone Surface/Volume
150
100
50
0
F
p = 0.6600
Trabecular Separation (μm)
0.4
0.3
0.2
0.1
0.0
G
p = 0.7247
Trabecular Number
4
3
2
1
0
H
p = 0.5549
Trabecular Pattern Factor
I
p = 0.4927
Centroid X (μm)
2.0
1.5
1.0
0.5
0.0
J
p = 0.1514
Centroid Y (μm)
K
p = 0.4213
Centroid Z (μm)

Fig. S7. Micro-CT analysis.

Micro-CT determinations of **A.** Bone volume, **B.** Bone/tissue volume ratio, **C.** Bone surface density, **D.** Bone surface, **E.** Bone surface/volume ratio, **F.** Trabecular separation, **G.** Trabecular number, **H.** Trabecular pattern factor, **I.** Centroid X, **J.** Centroid Y and **K.** Centroid Z for tibias from *Eμ-Myc; Rock1 WT* (n = 7) or *Eμ-Myc; Rock1 NC* (n = 5) mice. All *p* values were determined by unpaired Student's *t*-tests between the indicated groups. All graphs show means ± standard deviation, with data points representing individual mice ≤ 8 weeks of age.

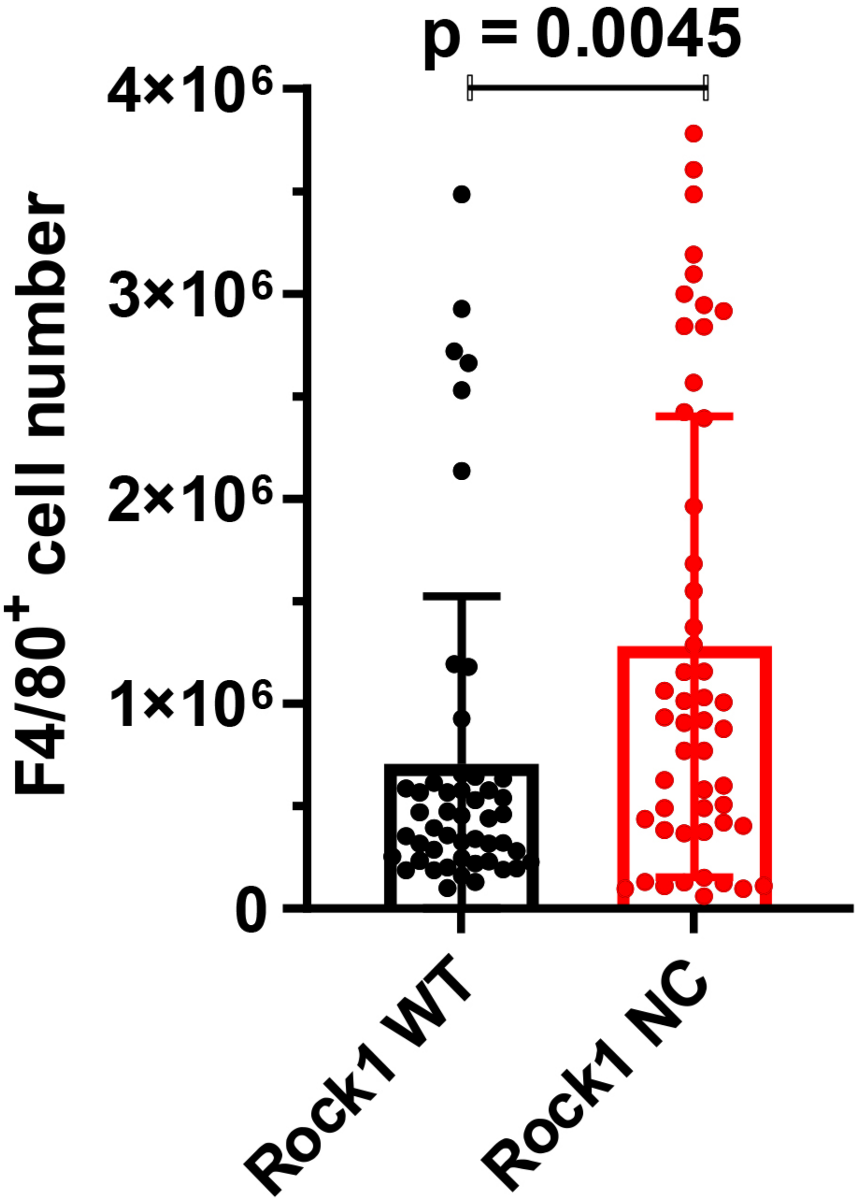

Fig. S8. Macrophages in *Rock1 WT* and *Rock1 NC* thymi.

Thymi from *Rock1 WT* (n = 50) and *Rock1 NC* (n = 51) mice aged between 4-8 weeks were harvested and cells were dissociated. Sorting for live single cells, followed by CD45, CD11B$^+$ and F4/80 $^+$ gave the total number of macrophages per thymus. Graph shows means ± standard deviation with data points representing individual mice. *P* value was determined by unpaired Student's *t*-test.

Disease Models & Mechanisms • Supplementary information

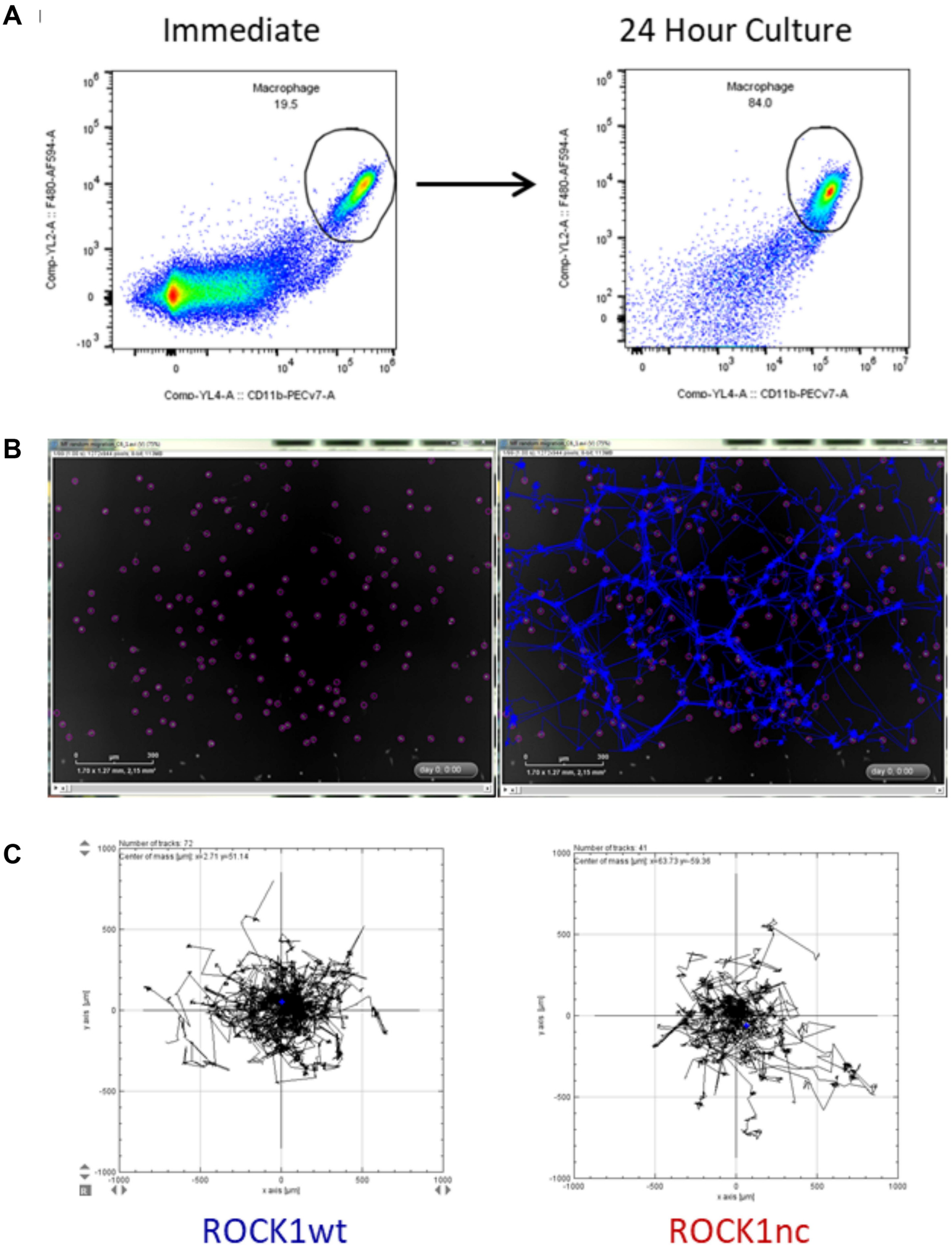

Disease Models & Mechanisms • Supplementary information

Fig. S9. Isolation, enrichment and characterization of macrophages from mouse peritoneum.

A. Mouse peritoneal washout plated in complete medium and cultured for 24 hours. Non adherent cells were aspirated leading to enrichment of adherent cells. Representative flow cytometry plots showing initial peritoneal isolate (left) and subsequent increase in percentage of cells identified as $CD11b^+$ $F4/80^+$ macrophages following 24 hours culture (right). **B.** Representative images showing identification of cells and generation of tracks in Trackmate Image J plugin, large and spread cells identified from tracking, and smaller rounded cells excluded. **C.** Representative spider plots generated in Image J Chemotaxis tool plugin, with data generated from Trackmate plugin showing no clear difference in random migration patterns of *Eμ-Myc; Rock1 WT and Eμ-Myc; Rock1 NC* macrophages.

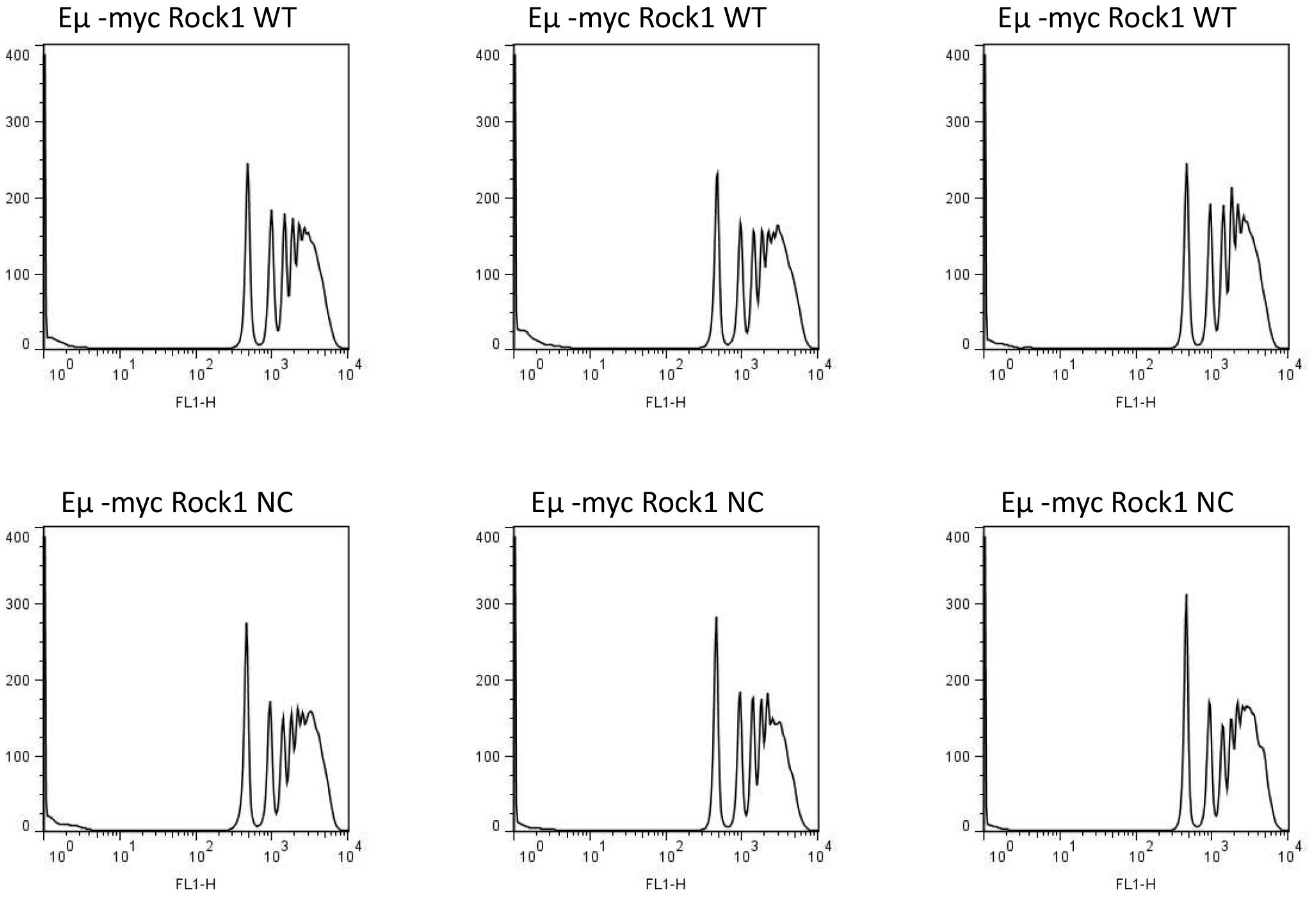

Fig. S10. Flow cytometry analysis of FluoSphere numbers in primary macrophages from *Eμ-Myc; Rock1 WT* and *Eμ-Myc; Rock1 NC* mice.

Representative flow cytometry profiles of macrophages isolated from 3 *Eμ-Myc; Rock1 WT* (upper panels) and 3 *Eμ-Myc; Rock1 NC* mice (lower panels) after 2 hours of incubation with opsonized FluoSphere beads.

Disease Models & Mechanisms • Supplementary information

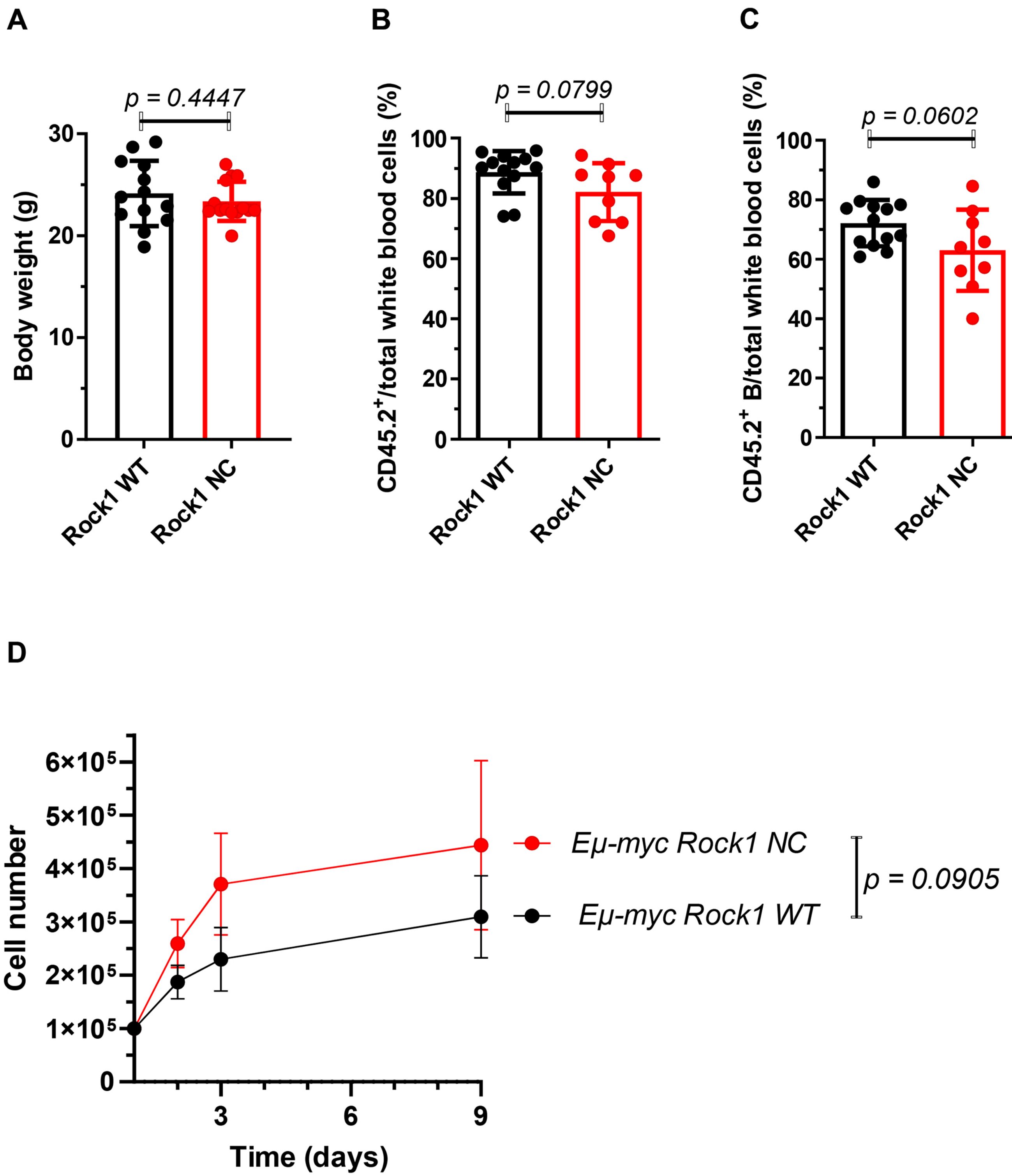

Disease Models & Mechanisms • Supplementary information

Fig. S11. Characterization of transplanted lymphoma cells.

Irradiated host *Rock1 WT* mice transplanted with *Eμ-Myc; Rock1 WT* (n = 13) or *Eμ-Myc; Rock1 NC* (n = 14) bone marrow were evaluated at experimental endpoint for **A.** Body weight, **B.** CD45.2$^+$/total white blood cell ratio and **C.** CD45.2$^+$ B cells/total white blood cell ratio. All *p* values were determined by unpaired Student's *t*-tests between indicated groups. All graphs show means ± standard deviation with data points representing individual mice.. **D**. Cell numbers at indicated days after plating for primary lymphoma cells isolated from *Eμ-Myc; Rock1 WT* (n = 13) or *Eμ-Myc; Rock1 NC* (n = 10) mice. Graph shows mean ± SEM. *P* value determined by two-way ANOVA and comparing the effect of mouse genotype as a source of variation.

Table S1. Hematology analysis.

Blood smear report	Incidence		Fisher's exact test *p* value
	WT	NC	
Polychromasia	1/7	2/7	$p > 0.9999$
Red blood cell spiculing	4/7	6/7	$p = 0.5594$
Hemoglobin crystals	2/7	3/7	$p > 0.9999$
Large lymphoid cells	1/7	0/7	$p > 0.9999$
Pyknotic cells	0/7	2/7	$p = 0.4615$

For hematology analysis, blood from each of 7 each of pre-neoplastic (≤ 8 weeks) *Eμ-Myc; Rock1 WT* and *Eμ-Myc; Rock1 NC* were analyzed. Polychromasia is an abnormally high number of red blood cells found in the bloodstream as a result of being prematurely released from the bone marrow during blood formation. Red blood cell spiculing may be present with liver or thyroid conditions. Hemoglobin crystals may be due to an enzyme deficiency such as G6PD, unstable hemoglobin variant, thalassemia, or autoimmune haemolytic anemia. Pyknosis is an irreversible condensation of chromatin in the nucleus of cells undergoing necrosis or apoptosis.

Table S2. Flow cytometry antibodies

Antibody	Supplier	Clone	Dilution	Fluorophore (catalogue number: RRID)		
B220	Biolegend	RA3-6B2	1/200	PerCPCy5.5 (103209: AB_312994)	APC (103211: AB_312996)	FITC (103205: AB_312990)
CD115	Biolegend	AFS98	1/100	BV421 (135513: AB_2562667)		
CD11b	Biolegend	M1/70	1/200	BV570 (101233: AB_10896949)	PE (101207: AB_312790)	APC (101211: AB_312794)
CD11c	Biolegend	N418	1/200	BV711 (117349: AB_2563905)	APC (117309: AB_313778)	
CD150	Biolegend	TC15-12F12.2	1/100	PECy7 (115913: AB_439796)		
CD19	Biolegend	6D5	1/200	PE (115507: AB_313642)	FITC (115505: AB_313640)	
CD206	Biolegend	C068C2	1/100	PECy7 (141719: AB_2562247)	BV605 (141721: AB_2562340)	PE (141705: AB_10896421)
CD25	Biolegend	PC61	1/100	AF700 (102024: AB_493709)		
CD3	Biolegend	17A2	1/200	FITC (100203: AB_312660)	APC (100235: AB_2561455)	
CD4	eBioscience	RM4-5	1/200	SB600 (63-0042-82: AB_2637461)	APC (17-0042-82: AB_469323)	
CD45	Biolegend	30-F11	1/200	BV510 (103137: AB_2561392)	BV421 (103133: AB_10899570)	AF700 (103127: AB_493714)
CD45.1	Biolegend	A20	1/200	BV421 (110731: AB_10896425)		
CD48	Biolegend	HM48-1	1/1000	BV711 (103439: AB_2650824)	FITC (103403: AB_313018)	
CD8	Biolegend	53-6.7	1/200	PECy7 (100721: AB_312760)	AF700 (100729: AB_493702)	APC (100711: AB_312750)
CD80	Biolegend	16-10A1	1/100	FITC (104705: AB_313126)		
CD86	Biolegend	GL1	1/100	AF700 (105023: AB_493720)	PerCP (105025: AB_893419)	APC (105011: AB_493343)
cKit	Biolegend	2B8	1/100	AF594 (105831: AB_2616614)	AF488 (105815: AB_493473)	PerCP (105821: AB_893230)
Clec4f	Biolegend	3E3F9	1/100	AF647 (156803: AB_2814081)		
F4/80	Biolegend	BM8	1/100	Biotin (123105: AB_893499). PE Streptavidin	AF594 (123140: AB_2563241)	PerCP (123125: AB_893495)
FoxP3	Biolegend	MF14	1/100	AF647 (126407: AB_1089116)		
Gr-1	Biolegend	RB6-8C5	1/250	APC (108411: AB_313376)		
IgD	Biolegend	11-26c.2a	1/250	APC (405713: AB_10645480)		
IgM	Biolegend	RMM-1	1/100	PECy7 (406513:		

Disease Models & Mechanisms • Supplementary information

				AB_10640069)		
Ki67	eBioscience	SolA15	1/100	Biotin (13-5698-82: AB_2572794). Streptavidin BV711		
Ly6C	Biolegend	HK1.4	1/400	PECy7 (128017: AB_1732093)		
Ly6G	Biolegend	IA8	1/200	AF700 (127621: AB_10640452)	APC (127613: AB_1877163)	
MHCII	Biolegend	M5/114.15.2	1/500	FITC (107605: AB_313320)	BV510 (107635: AB_2561397)	PE (107607: AB_313322)
NK1.1	Biolegend	PK136	1/200	BV711 (108745: AB_2563286)		
PDCA1	Biolegend	927	1/100	PerCPCy5.5 (127021: AB_2566646)		
Sca-1	Biolegend	D7	1/250	PE (108107: AB_313344)		
Siglec H	Biolegend	551	1/100	APC (129611: AB_10643574)		
TCRβ	Biolegend	H57-597	1/100	BV421 (109229: AB_10933263)		
TCRγδ	Biolegend	GL3	1/100	Biotin (118103: AB_313827). Streptavidin AF594		
Ter119	Biolegend	TER119	1/200	APC (116211: AB_313712)		

Abbreviations: AF = Alexa Fluor. APC = Allophycocyanin. BV = Brilliant Violet. CY = Cyanine. FITC = Fluorescein isothiocyanate. PE = Phycoerythrin. PerCP = Peridinin-Chlorophyll-Protein. SB = Super Bright

Disease Models & Mechanisms • Supplementary information

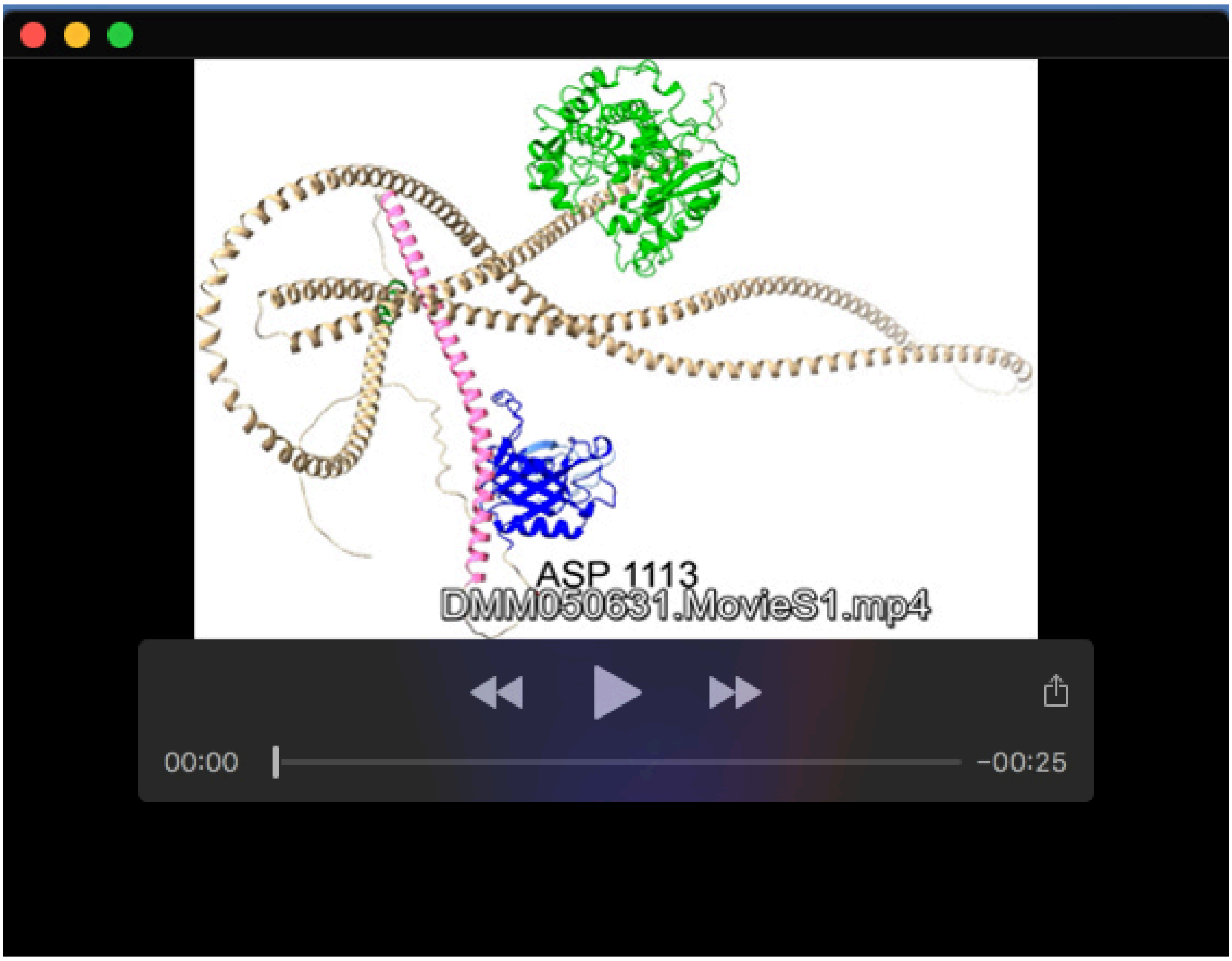

Movie 1. Predicted AlphaFold structure of ROCK1.

The AlphaFold (Jumper et al., 2021) predicted structure of full-length mouse ROCK1 (AF-P70335-F1). Kinase domain indicated in light green, pleckstrin homology domain indicated in dark blue, cysteine-rich domain indicated in light blue, rho-binding domain indicated in dark green, Asp1113 indicated in red. Image rendered and annotated with UCSF Chimera (Pettersen et al., 2004).
